# Clinical Evaluations Have Low Sensitivity for Identifying Preterm Infants in a Clinical Trial in a Limited Resource Setting

**DOI:** 10.1177/2333794X19857402

**Published:** 2019-06-25

**Authors:** Andrea G. Buchwald, Ibrahima Teguete, Moussa Doumbia, Fadima C. Haidara, Flanon Coulibaly, Fatoumata Diallo, Samba O. Sow, William C. Blackwelder, Milagritos D. Tapia

**Affiliations:** 1 Center for Vaccine Development and Global Health, University of Maryland School of Medicine, Baltimore, MD, USA; 2 Hôpital Gabriel Touré, Bamako, Mali; 3 Centre pour le Developpement des Vaccins—Mali, Bamako, Mali; 4 University of Maryland School of Medicine, Baltimore, MD, USA

**Keywords:** gestational age, New Ballard Score, preterm, fundal height, clinical trial

## Abstract

Preterm birth is a primary outcome of interest in maternal vaccination trials but determination of gestational age is challenging in limited-resource settings. This study compares the New Ballard Score and fundal height measurements with the current standard of early ultrasound for sensitivity of predicting preterm birth. A trial of maternal influenza vaccination was conducted in Bamako, Mali. The New Ballard Score and fundal height were collected on 4038 infants born in the trial, ultrasound data were available for 1893 of those infants. New Ballard Score and fundal height were compared, consecutively, to all ultrasound results, early ultrasound results from the first trimester, and the date of last menstrual period for estimation of gestational age. Sensitivity of the New Ballard Score for identifying preterm infants was 0.33 compared with early ultrasound and 0.1 compared with the last menstrual period based estimates of gestational age. Sensitivity of low birth weight alone was 0.43 compared with early ultrasound. New Ballard Score estimated gestational age within 1 week of ultrasound more frequently than fundal height (53% compared with 7.6%, respectively) yet New Ballard Score identified few infants as preterm (1.8% vs 5.8% by early ultrasound), and was biased toward categorizing low birth weight infants and infants requiring hospitalization as preterm. New Ballard Score is not an ideal measure for identifying preterm births in low-resource settings. Despite the time and cost of training required for correct measurement of New Ballard Score, measurement of low birth weight alone performed better than New Ballard Score for identifying preterm infants.

## Background

Maternal vaccination is a promising method for decreasing morbidity and mortality in young infants in low-resource settings.^1-4^ A primary safety outcome of interest in vaccine trials in pregnant women is the effect of maternal immunization on preterm births and small for gestational age (SGA).^5^ Accurate determination of gestational age (GA) is necessary to discriminate between preterm and SGA in newborns as both are important predictors of neonatal health outcomes. However, clinical issues differ between SGA and preterm infants and knowledge of GA is important to guide clinical decision making for newborn risk management.^6^ Low birth weight (LBW) is a common problem in low-resource settings and serves as a predictor of early-life morbidity and mortality due either to preterm birth or being SGA.^6,7^

Prediction of GA is difficult in any setting and a topic of substantial debate.^8-13^ While an imperfect measure, an ultrasound (US) conducted early in pregnancy is considered the most accurate method for estimating GA and is the current standard.^14-16^ In low-resource settings, US is not widely available and women commonly do not seek care until late in pregnancy. The date of the last menstrual period (LMP) is also used to estimate GA and is considered accurate when known. However, this information is often inaccurate due to poor recall, especially in women with lower education and socioeconomic status who are also more likely to delay seeking maternal care.^11,17-19^ The simplest and most widely available estimate of GA is based on fundal height (FH) measurements—standard of care for estimating expected date of delivery when US and LMP are unavailable.^20^ While it can provide a rough estimate for date of delivery, the estimate is imprecise and thus the utility of FH for determining SGA or preterm birth is considered limited.^20,21^

The Ballard method estimates GA of a newborn infant based on neurological and physical developmental characteristics; it is the standard of care in many low-resource settings where US and accurate LMP are largely unavailable.^8^ Despite relative ease of use, the assessment is complex, requiring trained staff to complete. In a large study in the United States, the Ballard method frequently overestimated the GA of preterm infants and underestimated the GA of postterm infants when compared with known LMP.^22^ Studies done in low-resource settings comparing GA based on the Ballard with LMP have yielded widely varying results and few studies have compared the Ballard method with US in a low-resource setting.^11,23-29^

We conducted a maternal influenza vaccine trial in 4193 pregnant Malian women from 2011 to 2014 and measured New Ballard Scores (NBS) and FH as well as recorded LMP and US results when available. The current study aims to evaluate the performance of the NBS and FH (standard of care) for estimating GA and identifying preterm infants in comparison with US and known LMP, in a low-resource setting under field conditions. Sensitivity, specificity, positive predictive value, and mean difference in GA estimations of clinical evaluations are estimated.

## Methods

### Study Design

As previously described, women believed to be in their third trimester of pregnancy were recruited from among women receiving prenatal care at health centers in Bamako, Mali.^30^ According to the Demographic Health Surveys, 95% of births in the population take place within community health centers.^31^ Women were enrolled according to the inclusion and exclusion criteria for the vaccine trial (described in detail in Tapia et al^30^). In brief, participants were included if they were in their third trimester of pregnancy, were able to understand and comply with study procedures, and intended to reside in the study area until newborn infants were 6 months old. Participants were excluded if another member of their household had already enrolled in the study, they had a history of severe reaction to vaccines, known active chronic infection (HIV, hepatitis B virus [HBV], and hepatitis C virus [HCV]), or known complications with the ongoing pregnancy.

All participants had uterine height measurements taken at enrollment to the study. US results were recorded from reports of US performed on women prior to enrollment or obtained after inclusion due to clinical indications. FH was measured at enrollment by study staff as described.^32^ Early US was defined as an US conducted at 15 weeks GA or earlier. Doctors and nurses were trained in conducting the Ballard scoring examination by an experienced neonatologist from the University of Maryland. Training was repeated 4 times throughout the study to reinforce understanding of the method and ensure consistency. NBS was obtained within the first 7 days of life using the new Ballard method.^8,23^ Study staff performing the NBS may have had knowledge of GA estimates obtained earlier in the pregnancy. Newborn weight, length, and head circumference were also measured at birth.

### Statistical Methods

All live-born infants from the study were included in descriptive analysis. A preterm infant was defined as any infant with an estimated GA less than 37 weeks by either US or LMP. A newborn was classified as preterm by NBS if the NBS score was 30 or less. Descriptive statistics were calculated for all newborns with GA measures. Among newborns with GA estimated by either US or LMP, estimates of sensitivity, specificity, and positive predictive value relative to US and LMP were calculated, and McNemar’s test for paired samples was used to compare the proportions defined as preterm between methods. Pearson correlation coefficients and matched-pair *t* tests were used to compare the estimated GA (in days) from early US (conducted ≤15 weeks GA), any US and LMP to the GA estimated by NBS and FH. Twins were excluded from all analyses using FH measurements, and only one twin from any twin set (chosen at random) was included in correlation calculations in order to avoid over-estimation of correlation.

Analysis was done using SAS 9.3 (SAS Institute, Cary, NC). Results with *P* < .05 were considered statistically significant.

### Ethics Approval and Informed Consent

Approval for the research was obtained from the University of Maryland, Baltimore Institutional Review Board (Approval Number HP-00049582); the ethics committee of the Faculté de Médecine, Pharmacie et Odonto-Stomatologie of Mali; and the Ministry of Health of Mali. Community sensitization was achieved through community leaders, health center representatives, and community members who attended community-wide meetings. All participants provided written informed consent. If the participant was illiterate, consent was obtained in the presence of a literate witness after listening to the audiotaped version of the consent form in Bambara, the local language. Participants who were illiterate placed their mark on the signature line and an independent literate witness signed and dated the form.

## Results

There were 4159 births in the maternal influenza vaccine trial, 54 of which were stillbirths, leaving 4105 live births to 4036 women for analyses. One individual was excluded due to data errors. Maternal characteristics of mothers of newborns included in the analysis are presented in Table 1. All participants were Sub-Saharan African. The distribution of GA varied by the method used to estimate GA, with FH estimating a lower mean GA as well as a wider range than all other methods (Table 2). FH classified a greater number of newborns as preterm than any other method and NBS classified fewer newborns as preterm than other methods (Table 2). Among newborns with GA calculated from US, 36% of LBW newborns were classified as preterm. Only 19% of LBW newborns were classified as preterm by NBS. Among newborns classified as preterm by NBS, 90% were LBW (Table 2).

**Table 1.** Characteristics of 4036 Women Who Gave Birth to Live Infants by Method of GA Determination.

Maternal Characteristics	Method Used to Calculate GA		
	US^a^ (n = 1882)	LMP^a^ (n = 262)	NBS or FH Only (n = 2047)
Age, mean (SD)^b^	24.7 (5.9)	25.2 (5.7)	24.7 (6.0)
Parity, mean (SD)^b^	3.0 (2.0)	2.6 (1.8)	3.5 (2.1)
Education level^b,c^			
None, n (%)	634 (33.4%)	32 (12.2%)	1117 (54.9%)
Elementary or Koranic, n (%)	965 (50.9%)	126 (48.1%)	839 (41.3%)
Finished secondary or higher, n (%)	296 (15.6%)	104 (39.7%)	75 (3.7%)
Household floor material			
Unfinished, n (%)^b^	162 (8.6%)	16 (6.1%)	307 (15.0%)
Finished, n (%)	1717 (91.4)	245 (93.9%)	1737 (85.0%)

Abbreviations: GA, gestational age; US, ultrasound; LMP, last menstrual period; NBS, New Ballard Score; FH, fundal height; SD, standard deviation.

a US and LMP categories are nonexclusive, 155 women both had an US completed and knew the date of their LMP.

b Indicates *P* < .05 by 2-sided *t* test (continuous variables), or by χ^2^ test (categorical variables).

c Thee participants are missing values for education level.

**Table 2.** Distribution of GA and Infants Classified as Preterm by Method Used to Determine GA^a^.

Method Used	N	Mean GA (Weeks)	SD	Range	<37 Weeks, N (%)	<2500 g, n (%)	LBW and Preterm,^b,c^ n (%)
All US	1917	39.5	1.7	29.3-45.1	129 (6.7)	151 (7.9)	55 (36.4)
Early US	633	39.5	1.6	32.4-45.1	37 (5.8)	53 (8.4)	16 (30.2)
LMP	263	39.6	2.1	33.4-45.6	30 (11.4)	20 (7.6)	6 (30.0)
FH^d^	3965	37.4	3.4	26.1-48.9	1692 (42.7)	267 (6.7)	150 (56.2)
NBS	4038	39.9	1.1	31.6-44.0	73 (1.8)	349 (8.6)	66 (18.9)

Abbreviations: GA, gestational age; SD, standard deviation; LBW, low birth weight; US, ultrasound; LMP, last menstrual period; FH, fundal height; NBS, New Ballard Score.

a Infants classified as preterm if GA estimated less than 37 weeks by method.

b Low birth weight infants are less than 2500 g.

c Number and percent of LBW infants classified as preterm.

d Twins excluded from all analyses with FH.

The distribution of GA varied by the method used to estimate GA, with FH estimating a lower mean GA as well as a wider range than all other methods (Table 2). FH classified a greater number of newborns as preterm than any other method and NBS classified fewer newborns as preterm than other methods (Table 2). Among newborns with GA calculated from US, 36% of LBW newborns were classified as preterm. Only 19% of LBW newborns were classified as preterm by NBS. Among newborns classified as preterm by NBS, 90% were LBW (Table 2).

The primary aim of this article was to determine the accuracy of NBS for estimating GA in a low-resource setting. US and LMP are used as standards for comparison. Newborns were included in further analysis only if they had a GA calculated either from US or LMP. There were 1917 newborns with a GA calculated from US; among newborns with US results, 156 had LMP measured. An additional 107 newborns had GA calculated from LMP but not from US. One newborn was excluded due to data errors, leaving a final study population of 2023 newborns.

Sensitivity of NBS for identifying preterm newborns was low, ranging from 10% to 33% depending on which comparator was used as the standard for comparison (Table 3). The proportion of newborns preterm by NBS was significantly different from the proportion of newborns preterm by both US and LMP. NBS was highly specific for identifying preterm infants and had high positive predictive values (PPVs) ranging from 75% to 92% (Table 3). FH had higher sensitivity for identifying preterm infants than NBS. However, FH had low PPV (05% to 14%) in all comparisons (Table 3). The proportion of newborns identified as preterm by FH was significantly different from the proportions identified by both US and LMP (Table 3).

**Table 3.** Sensitivity, Specificity, and PPV of NBS and FH for Identifying Preterm Infants^a^.

Method Used	Standard	N	Sensitivity	Specificity	PPV	*P* ^b^
NBS	All US	1893	0.242	0.998	0.912	<.01
	Early US	629	0.333	0.998	0.923	<.01
	LMP	255	0.100	0.996	0.750	<.01
Fundal height^c^	All US	1846	0.970	0.2919	0.072	<.01
	Early US	608	0.960	0.298	0.055	<.01
	LMP	257	0.857	0.341	0.137	<.01

Abbreviations: PPV, positive predictive value; NBS, New Ballard Score; FH, fundal height; US, ultrasound; LMP, last menstrual period.

a NBS and FH compared with all US, early US, and LMP.

b *P* value calculated for difference between proportions of infants estimated as preterm (<37 weeks) using McNemar’s test.

c Twins excluded from all analyses with FH.

### Correlation and Exact Agreement

Pearson’s correlation coefficient for GA by NBS compared with GA by US was 0.40 and did not change substantially when restricted to newborns with early USs. Correlation between NBS and LMP was lower (*r* = 0.25) and FH had higher correlation overall (*r* = 0.47 and *r* = 0.37, respectively). NBS estimated GA consistently closer to the standard than did FH. Over 50% of NBS estimates were within 1 week of US estimates and roughly 80% of all NBS estimates were within 2 weeks of US estimates (Table 4).

**Table 4.** Mean Difference in Estimates of GA and Fraction Within 1/2 Weeks of US Estimate.

Method Used	Comparison	N	MD (Days)	SE	≤1 Week Difference^a^	≤2 Weeks Difference^a^	Max Difference (Days)
New Ballard Score	All US	1893	8.57^b^	11.0	1002 (53%)	1538 (81%)	50.0
	Early US	629	8.16^b^	10.6	348 (55%)	516 (82%)	50.0
	LMP	255	10.72^b^	14.4	119 (47%)	185 (73%)	57.0
Fundal height^c^	All US	1,846	30.25^b^	0.544	140 (7.6%)	325 (18%)	96.8
	Early US	608	29.00^b^	0.90	51 (8.4%)	114 (19%)	77.8
	LMP	257	30.74^b^	1.55	22 (8.6%)	52 (20%)	79.0

Abbreviations: GA, gestational age; US, ultrasound; MD, mean difference; SE, standard error; LMP, last menstrual period.

a Proportion of New Ballard score and fundal height estimates of GA within 1 and 2 weeks of US and LMP estimates.

b Indicates *P* < .001 in matched-pair *t* test.

c Twins excluded from all analyses with fundal height.

There were 145 newborns who required hospitalization among our study population. Of these newborns, 80 had GA estimated by US, and 27 had GA less than 37 weeks by US. Of the 80 newborns with US measurements, 33.7% were classified as preterm by US, and they accounted for 21.0% of all newborns who were classified as preterm by US. One hundred forty-four newborns who required hospitalization had GA estimated by NBS; of these, 49 (34.0%) were classified as preterm by the NBS. Hospitalized newborns accounted for 70.0% of all newborns estimated as preterm by the NBS (Table 5).

**Table 5.** Proportion of Infants Classified as Preterm and That Required Hospitalization.

Method Used	Classified Preterm, N (% of Total)	Requiring Hospitalization, N (% of Total)	Preterm and Requiring Hospitalization, N^a^	% of Preterm Infants Requiring Hospitalization	% of Hospitalized Infants Classified as Preterm
All US	129 (6.7)	80 (4.2)	27	21.0%	33.7%
Early US	37 (5.8)	35 (5.5)	12	32.4%	34.3%
LMP	30 (11.4)	8 (3.0)	2	6.7%	25.0%
NBS	70 (1.7)	144 (3.6)	49	70.0%	34.0%

Abbreviations: US, ultrasound; LMP, last menstrual period; NBS, New Ballard Score.

a Infants who required hospitalization and were classified as preterm by each method.

## Discussion

In this population, NBS showed limited efficacy for estimation of GA. NBS identified a significantly smaller proportion of the population as preterm compared with US and had low specificity for identifying preterm infants. NBS estimates of GA had low overall correlation with US and LMP estimates, despite 80% of NBS estimates falling within 2 weeks of the US estimates. This is consistent with previous findings indicating that NBS biases toward estimating newborns at term with a 21.4% sensitivity for identifying preterm infants.^28^

In a small study in Gambia including 80 women, estimates of GA from NBS were highly inaccurate when compared with estimates from first term US, and NBS overestimated the number of preterm infants.^29^ A study in 364 Zimbabwean women reported high correlation of GA estimates by NBS and LMP when Ballard scores were adjusted by infant birth weight.^24^ Despite high correlation, the average error of a single observation in the Zimbabwe study was 1.89 weeks, indicating less accuracy than we found in Mali. Studies in non-African populations have had similarly varied results, but multiple studies have found that the NBS overestimates GA for preterm infants and has low sensitivity for identification of preterm infants.^22,23,25-27^ Extensive training of health care professionals on the use of NBS did not improve the performance of NBS in this population. In spite of repeated training in the use of the Ballard method conducted throughout the trial in order to improve the consistency, our study confirmed previous findings of low sensitivity for preterm infants.

Although NBS had low sensitivity for identifying preterm infants overall, our results agree with previous findings that the NBS is more accurate at identifying unhealthy newborns than healthy newborns as preterm.^26^ Clinical signs of illness appear to have influenced determination of GA by NBS, as newborns classified as preterm by NBS had a disproportionate number of hospitalizations compared with all other GA estimation methods. Similarly, LBW appears to bias NBS determination, as 90% of all newborns classified as preterm by NBS were LBW. LBW is sometimes used as a surrogate measure for preterm birth but can also be indicative of decreased fetal growth or SGA and is thus considered an inadequate marker for GA.^7^ In this study, LBW alone identified a greater proportion of newborns who were preterm by US than the proportion of newborns correctly identified as preterm by NBS. Our data suggest that the Ballard scoring system may do no better than birth weight alone for estimating GA.

While FH has shown limited utility for estimating GA in previous studies, it is standard of care in many low-resource settings and was measured for over 99% of singleton pregnancies in our study. By most comparisons, estimates of GA by FH fared worse than NBS estimates. The distribution of GA estimated by FH had high variability and less than 50% of GA estimates by FH were within 2 weeks of US estimates. Estimates of GA by FH had low PPV for identifying preterm infants. Our results confirm previous findings that FH can be useful for roughly predicting expected date of delivery in low-resource settings; however, the accuracy of FH in identifying preterm infants is not sufficient.^20^

The proportion of our study population classified as preterm was 5.8% by early US and 1.7% by NBS. Previous studies in West Africa and Mali have found rates of preterm birth from 5.3% to 13.3%.^33,34^ Women included in this study were provided with substantial monitoring and health education beyond the standard of care in Mali, which may have affected the rate of preterm births.

This study was limited by the lack of early US available for comparison; US estimates were restricted to newborns born to women who could afford the procedure. The population available for comparison and analyses had higher socioeconomic status in comparison to the individuals who had GA measured by only NBS or FH. Women of lower socioeconomic status would likely have a higher rate of preterm births; thus, if any bias exists, the proportion of newborns estimated as preterm by US should be underestimated.^35^ This bias does not change the interpretation of our results and confirms that NBS is underestimating the proportion of preterm infants in this population.

Ideally, this study would be done in a population where all women received US, regardless of socioeconomic status. Maternal vaccination trials in the future may need to develop novel methods to identify women earlier in pregnancy and should prioritize provision of US to participants, as enrollment of women only with US results would result in a less representative study population. Although the proportion of our population who had US done was low, this was the largest study done to date comparing US estimates to NBS estimates of GA, with the number of ultrasounds 10-fold higher than previous studies conducted in low-resource populations. A large population, in combination with the fact that the vaccine trial inclusion criteria introduced limited selection bias (women had to be relatively healthy to enroll in the trial), likely increases both the validity and generalizability of our findings concerning the accuracy of the NBS.

## Conclusions

The NBS had low sensitivity to identify preterm infants in a population of Malian newborns when compared with either US or LMP estimates of GA. The NBS preferentially detected preterm infants with LBW and clinical signs of illness related to preterm birth and overestimated the GA of preterm infants who appeared clinically healthy. Despite its low sensitivity for preterm births, NBS had much higher accuracy at predicting GA than FH, the current standard of care in low-resource settings. Measurements of FH had high variability between individuals and showed limited utility for estimating GA. Thus, while NBS may not be an ideal solution for the determination of GA in low-resource settings, it and other clinically determined methods of estimating GA may be preferable to reliance on FH. In circumstances where more precise estimates of GA are required, particularly in maternal vaccination trials where identification of preterm infants is an outcome of interest, substantial effort is needed to recruit women early in pregnancy and offer US in an unbiased fashion.
